# Clinical Holistic Medicine (Mindful, Short-Term Psychodynamic Psychotherapy Complemented with Bodywork) Improves Quality of Life, Health, and Ability by Induction of Antonovsky-Salutogenesis

**DOI:** 10.1100/tsw.2007.69

**Published:** 2007-03-02

**Authors:** Søren Ventegodt, Suzette Thegler, Tove Andreasen, Flemming Struve, Lars Enevoldsen, Laila Bassaine, Margrethe Torp, Joav Merrick

**Affiliations:** ^1^ Quality of Life Research Center, Teglgårdstræde 4-8, DK-1452 Copenhagen K, Denmark; ^2^ Research Clinic for Holistic Medicine, Copenhagen, Denmark; ^3^ Nordic School of Holistic Medicine, Copenhagen, Denmark; ^4^ Scandinavian Foundation for Holistic Medicine, Sandvika, Norway; ^5^ Interuniversity College, Graz, Austria; ^6^ Zusman Child Development Center, Soroka University Medical Center, Ben Gurion University of the Negev, Beer-Sheva, Israel; ^7^ National Institute of Child Health and Human Development, Jerusalem, Israel; ^8^ Office of the Medical Director, Division for Mental Retardation, Ministry of Social Affairs, Jerusalem, Israel

**Keywords:** chronic disease, family medicine, quality of life, CAM, short-term psychodynamic psychotherapy (STPP), holistic medicine, existential healing, bodywork, salutogenesis, Antonovsky

## Abstract

We had a success rate of treating low, self-assessed, global quality of life (measured by QOL1: How would you assess the quality of your life now?) with clinical holistic medicine of 56.4% (95% CI: 42.3–69.7%) and calculated from this the Number Needed to Treat (NNT) as 1.43–2.36. We found that during treatment, (in average 20 sessions of psychodynamic psychotherapy complemented with bodywork at a cost of 1600 EURO), the patients entered a state of Antonovsky-salutogenesis (holistic, existential healing), which also improved their self-assessed health and general ability one whole step up a 5-point Likert Scale. The treatment responders radically improved their self-assessed physical health (0.6 step), self-assessed mental health (1.6 step), their relation to self (1.2 step), friends (0.3 step), and partner (2.1 step on a 6-step scale), and their ability to love (1.2 step) and work (0.8 step), and to function socially (1.0 step) and sexually (0.8 step). It seems that treatment with clinical holistic medicine is the cure of choice when the patients (1) present the triad of low quality of life, poor self-assessed physical and/or mental health, and poor ability to function; and (2) are willing to suffer during the therapy by confronting and integrating old emotional problems and trauma(s) from the past. For these patients, the treatment provided lasting benefits, without the negative side effects of drugs. A lasting, positive effect might also prevent many different types of problems in the future. The therapy was “mindful” in its focus on existential and spiritual issues.

## INTRODUCTION

In the last 20 years, the concept of quality of life (QOL) has been the subject of intensive research and the medical establishment has used this concept more often as the primary goal for care and prevention. There are many different measures of QOL, from highly complex and compound measures, to the most simple one (QOL1) that asks the person to rate his own QOL today[1]. The last 2 decades have made the medical society expert in measuring QOL, so now we can easily say if a treatment improved a patent's global (total) QOL or not. Still, we miss the simple answer to the question on how to improve a patient's QOL. Sometimes, a very substantial intervention does not improve a patient's QOL, and sometimes a very subtle intervention changes the patient's whole life. We know that QOL can change dramatically and when this happens, even the most severe of somatic diseases seems to respond positively[2,3,4].

After studying 2000 factors related to eight different dimensions of QOL, we found that a positive philosophy of life seems to be the most important causal factor of global QOL[5,6,7,8,9] or, more precisely, the patient's consciousness seemed to be the primary determinant of QOL, health, and ability[10]. As a more positive philosophy of life in principle can be developed in any patient, in 1997 we started to work clinically to improve QOL and health with interventions on philosophy of life. Unfortunately, we did not succeed in this endeavor before we finally understood that negative philosophy of life was rooted in emotional traumas. When we started to work with psychodynamic short-term therapy, patients started to improve (by a few percent[11,12]), but when we added bodywork to give much more support to the patient's process of healing, we suddenly had a dramatic improvement of our results[13]. From the year 2000, we added bodywork and training in philosophy of life, and since then, more than 500 patients have been treated with increasing success.

## METHODS

The literature of psychodynamic short-term therapy demonstrated that much suffering could be cured when old emotional and existential problems were solved in therapy[14,15,16]. Bodywork added efficiency and speed to this method[17,18,19,20,21]. Philosophy of life gives direction and allows the patient to reach his or her goal with less deviation from the planned route of therapy. We define the combination of philosophy of life, psychodynamic short-term therapy, and bodywork as "clinical holistic medicine". This combination seems to be very much the same therapy — often called character medicine or humeral medicine — used in ancient Greece by Hippocrates (460–377 BCE) and his disciples on the island of Cos[22].

At our clinic, 55 patients with low or very low self-assessed QOL (measured with the validated questionnaire QOL1[1]) were treated with clinical holistic medicine (mindful, psychodynamic therapy with bodywork), inducing holistic healing (Antonovsky-salutogenesis[23,24]) from 2004–2005. All therapists had training in clinical holistic medicine from the Nordic School of Holistic Medicine, and the students also attended a European master program to improve their consciousness of themselves as therapists.

The purpose was to see if patients with a low global QOL could improve, if they healed their whole life, increasing their sense of coherence by use of Antonovsky-salutogenesis as proposed by Aaron Antonovsky (1923–1994)[23,24]. We expected all aspects of life to improve if the patients were able to heal their whole existence, and therefore we measured their QOL, health, and ability before and after the treatment. A total of 55 motivated patients entered the study and received clinical holistic medicine. For more details, please see Ventegodt et al.[13,25,26,27].

## RESULTS

We found self-assessed QOL low before treatment (bad or very bad) in 55 patients, self-assessed QOL high after treatment (very good, good, or neither good nor bad) in 31 patients, self-assessed QOL low after treatment (bad or very bad) in ten patients with a response rate at follow-up: nonresponders or dropouts: 14 patients, and self-assessed QOL low after treatment, nonresponders and dropouts: 24 patients (see Table 1).

**TABLE 1**
**Characteristics of Sample**

| | Before Treatment | After Treatment |
|---|---|---|
| Self-assessed QOL low | 55 | 10 |
| Self-assessed QOL high | 0 | 31; 31/55 = 56.36% (95%CI 42.32–69.70%)[33] |
| Nonresponders or dropouts | — | 14 |
| Self-assessed QOL low, nonresponder or dropout | 55 | 24 |

We had a success rate of treating low QOL of 31/55 = 56.4% (95% CI: 42.3–69.7%)[28] and calculated in this simplistic way, we found the Number Needed to Treat (NNT) of clinical holistic medicine with patients with low self-assessed QOL to be NNT = 1.43–2.36. We found the Number Needed to Harm to be NNH > 500 (estimated from more than 500 patients treated with no patients harmed).

Interestingly, we found that the patients had actually not only improved their self-assessed QOL (QOL1[1]) and their QOL measured with a more complex questionnaire (QOL5[1], see Table 4 and 5), but they had also dramatically improved their physical health; mental health; relation to self, friends, and partner; their ability to love and work; and to function socially and sexually (Tables 2 and 3). These last four questions combined with QOL1 and QOL5 make up QOL10. Most importantly, we found (using the square curve paradigm[29]) that these positive effects for the patients were lasting, since the effect of treatment had not significantly deteriorated 1 year later[13]. So, salutogenesis seemed to provide lasting benefits for the patients, without the side effects of drugs.

Tables 2 and 3 show that the 31 patients that healed existentially through salutogenesis and improved self-assessed QOL (QOL1[1]) also improved their self-evaluated physical and mental health as measured with QOL5[1], relationship with self, friends, and partner, ability to love, sexual ability of functioning, social ability and working ability. Please notice that the results are highly significant, both statistically and clinically.

If we look at their relations to self and other people (average of relation to self, partner, and friends), and their overall ability to function (average of love, work, sex, and social ability) these measures also improved. When we combined health, QOL, and ability in the combined measure QOL10, we also found that life as a whole had dramatically improved. This is the characteristic of salutogenesis. So we found that what happened for our patients was the healing of their existence and whole life.

Tables 4 and 5 show that the 31 patients who healed existentially also improved their relationships (with self, partner, and friends), their self-evaluated ability to function, and their QOL as measured with the validated questionnaire QOL5[1]. When health, QOL, and ability were combined, it is clear that these patients healed their whole life (as measured by QOL10[13]). This healing of all aspects of life is often seen with clinical holistic medicine and is called Antonovsky-salutogenesis after the researcher who discovered this kind of global healing of the patient's existence. The patients had, on average, about 20 sessions at a total cost of 1600 EURO.

**TABLE 2**
**Study of 31 Patients where Therapy Changed their Ratings of Self-Evaluated QOL from Low to Not Low* (T-Test)**

| | | Mean | N | Std. Deviation | Std. Error Mean |
|---|---|---|---|---|---|
| Physical health | Before | 2.900 | 30 | 0.9595 | 0.1751 |
| | After | 2.300 | 30 | 0.8366 | 0.1527 |
| Mental health | Before | 3.806 | 31 | 0.7491 | 0.1345 |
| | After | 2.193 | 31 | 0.7032 | 0.1263 |
| Self-esteem | Before | 3.451 | 31 | 0.8098 | 0.1454 |
| | After | 2.258 | 31 | 0.6815 | 0.1224 |
| Relation to friends | Before | 2.387 | 31 | 0.8032 | 0.1442 |
| | After | 2.064 | 31 | 0.7718 | 0.1386 |
| Relation to partner | Before | 4.548 | 31 | 1.8044 | 0.3240 |
| | After | 2.483 | 31 | 1.6905 | 0.3036 |
| Ability to love | Before | 3.580 | 31 | 1.0574 | 0.1899 |
| | After | 2.419 | 31 | 1.1187 | 0.2009 |
| Sexual ability | Before | 3.290 | 31 | 1.0390 | 0.1866 |
| | After | 2.483 | 31 | 1.0286 | 0.1847 |
| Social ability | Before | 3.096 | 31 | 0.9075 | 0.1630 |
| | After | 2.096 | 31 | 0.7463 | 0.1340 |
| Work ability | Before | 3.096 | 31 | 0.8700 | 0.1562 |
| | After | 2.322 | 31 | 0.7910 | 0.1420 |
| QOL | Before | 4.129 | 31 | 0.3407 | 0.0612 |
| | After | 2.193 | 31 | 0.7032 | 0.1263 |

* From 4 = bad or 5 = very bad, to 1 = very good, 2 = good, or 3 = neither good nor bad.

**TABLE 3**
**Paired Samples Test**

| | Paired Differences | | | | | t | df | Significance (Two–Tailed) |
|---|---|---|---|---|---|---|---|---|
| | Mean | Std. Deviation | Std. Error Mean | 95% CI of Difference | | | | |
| | | | | Lower | Upper | | | |
| Physical health | 0.6000 | 0.8550 | 0.1561 | 0.2807 | 0.9193 | 3.844 | 29 | 0.001 |
| Mental health | 1.612 | 1.0855 | 0.1949 | 1.214 | 2.011 | 8.272 | 30 | 0.000 |
| Self-esteem | 1.193 | 1.0776 | 0.1935 | 0.7983 | 1.588 | 6.167 | 30 | 0.000 |
| Relation to friends | 0.3226 | 0.8321 | 0.1494 | 0.0173 | 0.6278 | 2.158 | 30 | 0.039 |
| Relation to partner | 2.064 | 2.1899 | 0.3933 | 1.261 | 2.867 | 5.249 | 30 | 0.000 |
| Ability to love | 1.161 | 1.5512 | 0.2786 | 0.5923 | 1.730 | 4.168 | 30 | 0.000 |
| Sexual ability | 0.8065 | 1.3017 | 0.2338 | 0.3290 | 1.283 | 3.449 | 30 | 0.002 |
| Social ability | 1.000 | 1.0954 | 0.1967 | 0.5982 | 1.401 | 5.083 | 30 | 0.000 |
| Work ability | 0.7742 | 1.0865 | 0.1951 | 0.3756 | 1.172 | 3.967 | 30 | 0.000 |
| QOL | 1.935 | 0.8538 | 0.1533 | 1.622 | 2.248 | 12.62 | 30 | 0.000 |

**TABLE 4**
**Study of 31 Patients where Therapy Changed their Ratings of Self-Evaluated QOL from Low to Not Low* (T-Test)**

| | | Mean | N | Std. | Std. Mean |
|---|---|---|---|---|---|
| Relations | Before | 3.462 | 31 | 0.7634 | 0.1371 |
| | After | 2.268 | 31 | 0.7119 | 0.1278 |
| Ability | Before | 3.266 | 31 | 0.6121 | 0.1099 |
| | After | 2.330 | 31 | 0.5715 | 0.1026 |
| QOL (QOL 5) | Before | 3.416 | 30 | 0.5061 | 0.0924 |
| | After | 2.244 | 30 | 0.5685 | 0.1038 |
| Health-QOL-Ability (QOL 10) | Before | 3.353 | 30 | 0.4536 | 0.0828 |
| | After | 2.265 | 30 | 0.5262 | 0.0960 |

**TABLE 5**
**Paired Sample Test**

| | Paired Differences | | | | | t | df | Significance (Two–Tailed) |
|---|---|---|---|---|---|---|---|---|
| | Mean | Std. Deviation | Std. Error Mean | 95% CI of Difference Lower | 95% CI of Difference Upper | | | |
| Relations | 1.193 | 0.8596 | 0.1544 | 0.8782 | 1.508 | 7.730 | 30 | 0.000 |
| Ability | 0.9355 | 0.8802 | 0.1581 | 0.6126 | 1.258 | 5.917 | 30 | 0.000 |
| QOL (QOL 5) | 1.172 | 0.6829 | 0.1246 | 0.9172 | 1.427 | 9.401 | 29 | 0.000 |
| Health-QOL-Ability (QOL 10) | 1.088 | 0.6590 | 0.1203 | 0.8419 | 1.334 | 9.042 | 29 | 0.000 |

## DIISCUSSION

Holistic healing, improving all aspects of life at the same time, was predicted to be a possibility by Antonovsky[23,24]. It seems possible by rehabilitating a person's character, mission of life, and sense of coherence, and that is the aim of clinical holistic medicine[25,26,27]. We found the process possible for the patients who were prepared to assume responsibility for their own life, even when this meant confronting old emotional problems. Of 55 patients who started the study, 14 patients dropped out of the follow-up study and ten did not improve; 31 patients were helped to a QOL that was no longer bad or very bad. Of these, 20 had a good or very good QOL after treatment, and 11 rated their QOL as neither good nor bad.

According to the square curve paradigm[29], it has no meaning to use a placebo control when we use shifts in consciousness to induce Antonovsky-salutogenesis. What we need to do is to demonstrate that the process of healing is actually taking place in a very short time, to exclude any likelihood of the patients being helped any other way than by our treatment, and the patients must be chronically ill or in a similar chronically poor condition, so that they do not shift by themselves. We believe that both these conditions were fulfilled[13]. All the patients had been to their own general practitioner without getting the help they needed. About one in three of the patients had been to a psychiatrist, psychologist, or had psychopharmacological treatment before entering the study.

The most severe problem with this study is that our patients may be highly motivated for personal development because they were attracted to the clinic by books and other material that presented the idea of personal growth as a key to life's problems[25,26,27,30,31,32,33,34]. This makes it a relevant question whether a random sample of the Danish population with poor QOL would have done similarly well. Further research is needed to answer the import question of who can be helped this way.

## CONCLUSION

We had a success rate of treating low, self-assessed, global QOL (as measured with QOL1: How would you assess the quality of your life now?[1]) of 31/55 = 56.4% (95%CI: 42.3–69.7%); calculated in the most simplistic way. We found the NNT of clinical holistic medicine with patients with low, self-assessed QOL to be NNT = 1.43–2.36. We found the NNH > 500 (estimated from more than 500 patients being treated with no patients harmed).

We found that the patients had entered a state of salutogenesis (existential healing) and that in addition to their global QOL, they also improved their self-assessed health and ability about one step up the 5-point Likert Scale. The treatment responders dramatically improved their self-assessed physical and mental health; their relation to self, friends, and partner; their ability to love and work; and to function socially and sexually. In spite of having treated more than 500 patients, no patient has yet been harmed, indicating that the induction of Antonovsky's salutogenesis is an efficient and least dangerous method of healing we have today. Unfortunately, this cure is not yet for everybody, but only for the motivated patients who are willing to suffer during the therapy from confronting and integrating old emotional problems and traumas all the way back from early childhood. For the patients that are able to endure the therapy, the treatment seems to provide lasting benefits for the patients, without the side effects of drugs. The lasting, positive effects also seem to prevent many problems in the future.

## ACKNOWLEDGMENTS

This study was supported by grants from IMK Almene Fond. The quality of life research was originally approved by the Copenhagen Scientific Ethical Committee under number (KF)V.100.2123/91 and later correspondence.
